# Assessing the Clinical Relevance of *BRCA1* RING Domain Variants of Uncertain Significance

**DOI:** 10.3390/curroncol33070399

**Published:** 2026-07-03

**Authors:** Matthew D. Martin, Gabriella C. Torretto, Kaamraan Islam, Nicole E. Archer, Harriet E. Feilotter, Scott K. Davey

**Affiliations:** 1 Department of Pathology and Molecular Medicine, Queen’s University, Kingston, ON K7L 3N6, Canada; 18mdm7@queensu.ca (M.D.M.);; 2 Division of Cancer Biology and Genetics, Sinclair Cancer Research Institute, Queen’s University, Kingston, ON K7L 3N6, Canada; 3 Laboratory Medicine Program, University Health Network, Toronto, ON M5G 2C4, Canada

**Keywords:** hereditary breast cancer, hereditary ovarian cancer, BRCA1, variants of uncertain significance, germline genetic testing, machine learning, functional assays, The American College of Molecular Genetics

## Abstract

The BRCA1 protein serves an essential function in maintaining genomic integrity, to the extent that up to 80% of women carrying a pathogenic *BRCA1* variant develop breast cancer (BC). Most of these carriers would benefit from prophylactic care, but genetic screens that uncover variants of uncertain significance (VUSs) do not provide insight on disease risk or clinical decision-making. In accordance with guidelines established by The American College of Molecular Genetics (ACMG) and Association for Molecular Pathology (AMP), this study produced computational and functional evidence to inform the reclassification of *BRCA1* VUSs as pathogenic or benign, with a specific focus on the abundant subset of missense variants within the RING domain. A six-feature linear support vector machine (LSVM) specifically trained on BRCA1 RING variants performed well (84% accurate in predicting in vitro binding loss) and provided supporting classification evidence for 322 VUS. A mammalian cell co-immunoprecipitation (co-IP) assay that quantified the binding between variant BRCA RING constructs and endogenous BARD1 provided corroborating strong evidence for nine VUSs and correlated with a homology-directed repair (HDR) assay by Starita et al. (*p* = 0.04). The combined evidence warrants the reclassification of three VUSs as likely benign (N16S, A17D, and E100D) and one as likely pathogenic (H41P), and underscores the promise of domain-specific approaches for missense VUS reclassification.

## Simple Summary

Women who inherit harmful variants in the *BRCA1* tumour suppressor gene have an elevated risk of developing multiple cancers, and may benefit from increased screening and preventative surgeries. Unfortunately, ~40% of *BRCA1* variants have an unknown effect on cancer risk, leaving their carriers with inconclusive information to guide care decisions. This study provides evidence for the classification of variant effects within *BRCA1*’s RING region using two complementary approaches: (1) an artificial intelligence algorithm designed to predict the severity of variant impacts, and (2) a cell-based assay that evaluates whether variant BRCA1 RING proteins retain an interaction required for normal functioning. The results were generally consistent, and confidently clarified the effect of four variants on cancer risk. This study both aids the preventative care decisions for carriers of these variants and underscores the potential of protein-specific approaches for variant characterization.

## 1. Introduction

Inherited susceptibility factors underlie 5–15% of female BC and 10–25% of ovarian cancers (OCs) [1,2,3,4]. Many cases arise from Hereditary Breast and Ovarian Cancer Syndrome (HBOC), where germline variants increase the risk for early-onset multifocal carcinoma in diverse sex-steroid-responsive epithelial tissues [2]. High-penetrance variants in *BRCA1* and *BRCA2* tumour-suppressor genes have a low population prevalence, but account for 60–65% of HBOC-associated cancer [5,6,7]. Both proteins function in the HDR of double-stranded DNA breaks (DSBs), but evolutionary divergence results in distinct cancer spectra: pathogenic *BRCA1* variants cause a higher lifetime risk of female BC (60–80%) and OC (30–40%), while *BRCA2* is more relevant to multifocal and male cancers [8,9]. BRCA1 is relatively more pleiotropic within the DSB response, additionally functioning as the coordinating hub for upstream complexes that mediate damage detection, chromatin remodelling, and cell cycle arrest [9,10,11]. Amplified by cyclical proliferation-associated replication stress, *BRCA1* haploinsufficiency predisposes epithelial breast and ovarian cells to accumulate mutations that can drive carcinogenesis or enable survival despite the loss of heterozygosity at *BRCA1* [12,13,14]. Resulting from this genomic instability, tumours tend to be poorly differentiated [15,16], lack targetable receptors [17], and exhibit a dependence on PARP-mediated base-excision repair [18].

Screening programs to identify women carrying pathogenic *BRCA1* variants are valuable to guide early prophylactic intervention [19,20,21], enhanced surveillance [22,23,24], lifestyle and fertility counselling [25,26], and the cascade testing of relatives [27]. Conversely, screens with no variants or benign variants not associated with an increased cancer risk can alleviate anxiety in women referred due to a suggestive family history of HBOC [28]. In the context of established disease, pathogenic variants predict an increased sensitivity to platinum agents and synthetic lethal PARP inhibition [18,29], whereas benign variants are associated with a lower risk for HBOC-related second primary cancers [30]. Unfortunately, VUSs—variants for which the associated cancer risk is not confidently understood—account for 39% of the currently documented *BRCA1* variants, and 87% of missense variants. This finding does not afford cascade testing for relatives, and often confuses patients who are ultimately left to make care decisions based on the limited risk insight [31,32,33].

The widely adopted 2015 ACMG-AMP variant classification guidelines require ≥95% confidence for clinically actionable likely benign and likely pathogenic classifications [34]. The probability of variant pathogenicity is derived by integrating weighted evidence types (supporting < moderate < strong < very strong < stand-alone), with the strength approximately doubling at each successive tier. Reflecting the potential for a clinically consequential false-positive interpretation, the likely pathogenic classification carries a higher evidentiary burden to reach the shared confidence threshold. Approximately 70% of known pathogenic *BRCA1* variants are truncating and classified with very strong evidence of null allele production, which is not available to most missense variants outside of the initiator methionine and splice sites. Variants found within individuals and families who display a disease-related phenotype (hereditary or spontaneous cancer) can yield supporting to stand-alone evidence depending on the association strength, but analyses are often resource-intensive and infeasible for rare variants [35,36]. Nonetheless, their absence from the Genome Aggregation Database (gnomAD) is consistent with negative selection and provides moderate evidence of pathogenicity, as does the presence of a known pathogenic variant at the same residue. Variant effect predictors (VEPs) generate supporting evidence by computationally estimating the variant impact using allele frequency, biochemical change, evolutionary conservation, and cellular fitness consequences (fitCons) [37,38]. Ensemble approaches often employ machine learning to integrate these features and optimize the performance across diverse training variants, aiming for proteome-wide generalizability. Given the limited capture of protein-specific features relevant to variant effects, a single line of evidence should be informed by multiple VEPs that accurately discriminate between variant classes within the target protein [34]. In vitro assays observing the loss or retention of a disease-implicated protein function can provide strong evidence for and against variant pathogenicity, respectively. Interpretation is limited for pleiotropic BRCA1, as the functions strictly required for tumour suppression remain unresolved. While pathogenicity correlates with the disruption of HDR [39,40], centrosome regulation [41], transcription [42,43], localization [44], ubiquitination [45,46], and haploid cell viability [35], discordant results suggest that isolated function loss is insufficient to drive tumourigenesis due to redundancy or compensation.

Established BRCA1 pathogenic missense variants, along with VUSs identified in women with a family history suggestive of HBOC, cluster in the amino-terminal RING and carboxy-terminal BRCT, suggesting that each domain independently contributes an indispensable tumour-suppressive function. The intervening region is relatively unstructured, providing conformational flexibility that supports domain function through binding diverse ligands, and positioning termini to engage distal targets [47,48]. The interaction between BRCA1 RING and BARD1 RING appears critical for driving the overall domain activity. Previous publications have shown that BARD1 binding distinguishes between non-splice-altering pathogenic and benign BRCA1 RING variants with 100% accuracy [46,49]. Pathogenic variants are specifically enriched at critical residues for this interaction: zinc-coordinating residues (C24, C27, C39, H41, C44, C47, C61, and C64) within two binding loops that orient α-helix 1 (residues 7–22) and α-helix 3 (residues 80–97) to form an extensive BARD1 interface are most intolerant to substitution, with pathogenicity also associated with disruptions to interface hydrophobicity and binding loop elements that position zinc-coordinating residues, including α-helix 2 (residues 45–54), a three-stranded β-sheet (residues 35–36, 42–43, and 74–75), and proline backbone constraints [50,51].

BARD1 functions as a scaffold and modulator for BRCA1-associated complexes, notably enhancing the contralateral binding and activity of a ubiquitin-conjugating enzyme (E2) [52,53]. At least eleven E2 enzymes can bind individually, generating partly redundant signals that converge on genome stability and G1/S and G2/M checkpoint control, with UBE2D3 currently understood to have the broadest reactivity [54]. Interestingly, correlations between pathogenicity and centrosome amplification, HDR deficiency, and dysregulated gene expression are partly rationalized by the loss of the E2-mediated proteasomal degradation of γ-tubulin [55], histone H2A [56,57], and pro-growth transcription factors [58], respectively. Beyond effector functions, the BRCA1:BARD1 interaction buries the nuclear export sequences (NES; residues 22–30 and 81–99) to drive genomic localization [59,60], and may be essential for the overall protein stability and protection against CRL4-mediated degradation [61,62,63,64]. Further supporting functional dependence, the rarity of *BRCA1* + *BARD1* double heterozygotes is consistent with either synthetic lethality or the absence of an additional fitness benefit from a second mutation [65,66]. Moreover, phenotypic similarities between *BARD1*- and *BRCA1*-deficient cancers— including high mutation load, cell cycle dysregulation, and PARP inhibitor sensitivity—suggest that both deficiencies converge on similar pathway alterations driven by a common upstream loss in BRCA1:BARD1 interaction [67]. Similar characteristics are also observed in cancers expressing RING-less BARD1 isoforms, which act as competitive antagonists of BRCA1-associated complex formation [68].

Accordingly, a mammalian cell co-IP quantifying BARD1 binding capacity should serve as a proxy for overall BRCA1 RING domain activity and capture its indispensable tumour-suppressive function, providing strong classification evidence. An algorithm trained using BRCA1 RING variants should capture key features of pathogenicity within the domain, enabling robust supporting evidence and variant prioritization for in vitro analysis.

## 2. Materials and Methods

### 2.1. Building BRCA1 RING-Specific Computational Classifier

All (*n* = 632) clinically documented germline missense single nucleotide variants within the BRCA1 RING domain (residue 1–109) were obtained from ClinVar (v01/04/2024). Preprocessing excluded variants at the start codon (*n* = 10), R71 splice site (*n* = 8), and those with synonymous (*n* = 1) and convergent (*n* = 48) effects on amino acid sequence. ClinVar-curated evidence was evaluated to confirm ACMG-AMP-concordant classification and sort the 587 preprocessed variants into pathogenic (including likely pathogenic; *n =* 56), benign (including likely benign; *n =* 50), and VUS (*n =* 459) classes [34]. Variants were then evaluated on 52 computational VEP algorithms identified through an extensive literature search: 47 from the dbNSFPv4.0 database (Supplementary Table S1), BLOSUM62, PON-P2, BRASS NN, Align-GVGD with built-in Human-to-Urchin alignment, and Shannon Entropy using multiple sequence alignments (MSAs) built from mammalian and vertebrate NCBI Orthologs >1500 residues on COBALT (*n_vertebrate_* = 264, *n_mammalian_* = 133). Algorithms utilizing in vitro functional data were excluded to ensure independence from functional evidence, and splice algorithms were omitted since predictions do not reflect cDNA assay outcomes. Feature outputs were rescaled using min–max normalization to a [0, 1] range, with 0 corresponding to the algorithm-specific minimum (most benign prediction), and 1 to the maximum (most pathogenic).

Pathogenic and benign variants were split ~2:1 into training (38 pathogenic; 34 benign) and test (18 pathogenic; 16 benign) sets using positionally stratified random sampling to maximize coverage across the domain. Labelled and rescaled training variant scores across 52 VEP features were evaluated on the Molecular Feature Selection Tool (MolecularFeaST; Renwick Lab at Queen’s University; Kingston, ON, Canada) using 5-fold cross-validation [69]; feature rank and average discriminatory performance (measured as pixel height on output plot) were individually rescaled using min–max normalization from 0 (lowest-performing algorithm) to 1 (highest-performing). The inflection point of diminishing returns was defined at the largest residual between observed performance and a reference model representing a linear performance decline with decreasing rank (y = x). The set of features that performed above this inflection point were screened for redundancy—those used in the training of a higher-ranked ensemble feature were removed.

Remaining top-performing, non-redundant features were subject to backwards elimination to identify an optimal feature set. Labelled training variant scores were used to train all available models (*n* = 32) in the MATLAB^®^ Classification Learner Application (CLA; MATLAB R2024a; Mathworks^®^; Natick, MA, USA) with 5-fold cross-validation. The process was repeated with sets that iteratively removed the lowest-performing feature, and the group yielding the highest average training accuracy across all models was selected as the final feature set. The choice of a specific CLA model trained on these finalized features was guided by overall training and test accuracy, combined with insights from t-distributed stochastic neighbour embedding (t-SNE) plots on efficient decision boundary topology.

On the final model, supporting computational evidence thresholds were defined by the 95% confidence intervals of pathogenic and benign training and test variant outputs. Significance between pathogenic and benign variant scores was tested using a one-tailed Welch’s *t*-test.

Pathogenic and benign variants within other zinc-finger domains identified in the HUGO Gene Nomenclature Committee Index and UniProt (RING, PHD, C2HC, CXXC, TAZ, ZZ, and C2H2) were collected, preprocessed, and evaluated on a modified algorithm (non-generalizable features were removed) applying the same methodology.

### 2.2. Cloning Variant BRCA1 RING Constructs

Given the 459 BRCA1 RING VUS, algorithm outputs were used to prioritize variants for in vitro testing, enriching for VUSs with the highest-confidence predictions as they were expected to yield consistent and well-defined BARD1 binding results that would maximize low-throughput assay value. Moreover, mid-scoring VUSs with diverse structural and positional features were also selected for in vitro analysis to assess algorithm performance across the full output range. VUSs were grouped into three batches based on score: high (top 3%) and low (bottom 3%), intermediate (1.0 to −1.0), and moderate–high/–low (remaining range). Six controls were selected to capture the diversity of pathogenic (C24R, C44Y, and M18T) and benign (wildtype, D67Y, and K45R) BRCA1 RING variants.

BRCA1 RING double-stranded DNA gBlocks Gene Fragments were synthesized for each selected variant (Integrated DNA Technologies^TM^; Coralville, IA, USA), containing the human BRCA1 RING sequence (nucleotides 1–327) with a carboxy-terminal 1×HA tag, TAA stop codon, 5’*Bam*HI and 3’*Not*I restriction enzyme sites, and short random flanking sequences to ensure proper restriction enzyme cleavage. Lyophilized gene fragments were reconstituted in nuclease-free water at 20 ng/μL, cloned into pCR-Blunt II-TOPO plasmids using the Zero Blunt^®^ TOPO^®^ PCR Cloning Kit (Invitrogen^TM^, Thermo Fisher Scientific^TM^; Burlington, ON, Canada), transformed according to the One-Shot^®^ TOP10 Chemically Competent *E. coli* protocol (Invitrogen^TM^, Thermo Fisher Scientific^TM^), and plated for overnight growth at 37 °C on lysogeny broth + kanamycin at 50 μg/mL. Two colonies were selected and amplified overnight in 5 mL of the same media. Plasmid DNA was purified according to the QIAprep^®^ Spin Miniprep Kit protocol (QIAGEN^®^; Louisville, KY, USA), and digested using high-fidelity *Bam*HI and *Not*I in New England Biolabs^®^ (NEB^®^) rCutSmart buffer for 25 min (NEB^®^; Whitby, ON, Canada). Reactions were separated via 1.5% agarose electrophoresis at 100 V, and the QIAquick^®^ Gel Extraction Kit protocol (QIAGEN^®^) was used to excise and purify BRCA1 RING DNA (364 base-pairs). pTRE2 (Clontech, Takara Bio USA, Inc.; San Jose, CA, USA) was digested, extracted (3722 base-pairs), and purified according to the same protocol. T4 DNA Ligase (M0202) was used to ligate BRCA1 RING fragments and linearized pTRE2 according to NEB^®^ protocol. These pTRE2-RING plasmids were transformed into One-Shot^®^ TOP10 cells and cultured overnight at 37 °C on lysogeny broth + ampicillin (100 μg/mL). One colony was selected, amplified overnight in 5 mL of the same media, and then subject to the QIAprep^®^ Spin Miniprep Kit protocol to isolate target plasmid. Digestion with *Bgl*II/*Bam*HI/*Not*I in NEB^®^ rCutSmart buffer for 25 min, and Sanger sequencing (The Centre for Applied Genomics Sequencing Facility; Toronto, ON, Canada) confirmed successful cloning before GeneJet Plasmid Maxiprep Kit (Thermo Fisher Scientific^TM^) was used to amplify and isolate miniprepped pTRE2-RING plasmid.

### 2.3. Cell Culture and Co-IP

Wildtype (WT) and C24R variants were used as controls in all VUS batches. Human Embryonic Kidney 293 cells expressing SV40 large T-antigen (HEK293T, ATCC#: CRL-1573; RRID: CVCL_0063; obtained from Queen’s University Sinclair Cancer Research Institute, Kingston, ON, Canada) were prepared in high-glucose, 1% Na-pyruvate Dulbecco’s Modified Eagle Medium (Gibco^TM^, Thermo Fisher Scientific^TM^) supplemented with 10% Fetal Bovine Serum (Gibco^TM^, Thermo Fisher Scientific^TM^) and 1% Penicillin Streptomycin (Gibco^TM^, Thermo Fisher Scientific^TM^) at 37 °C in a 5% CO_2_ atmosphere. Two 10 cm plates were seeded for each variant (6 million cells, 10 mL media); once confluence reached 75%, pTRE2-RING plasmids were transfected using an optimized Lipofectamine^TM^ 3000 protocol with 1000 μL Opti-MEM, 21.7 μL L3000, 20 μg DNA, 28 μL P3000, and 15 min mixing time for each plate (Invitrogen^TM^, Thermo Fisher Scientific^TM^). Cells were incubated for 48 h before harvesting by trypsinization and centrifugation (760× *g*, 8 min, swing-bucket) with two 5 mL cold Phosphate-Buffered Saline (PBS; Gibco^TM^, Thermo Fisher Scientific^TM^) washes. Lysis was completed with 10 μL Pierce^®^ IP Whole-Cell Lysis Buffer (Thermo Fisher Scientific^TM^) substituted with 1% 100× Halt Protease Inhibitor Cocktail (Thermo Fisher Scientific^TM^) per million cells, following manufacturer instructions. Cell debris was collected by centrifugation at 4 (16,000× *g*, 10 min, fixed-angle), and Pierce^TM^ BCA Protein Assay Kit (Thermo Fisher Scientific^TM^) quantified protein concentration in whole-cell lysates (WCLs). A 20 μL WCL fraction was reserved and diluted in 1×SDS+2% β-mercaptoethanol (BME) sample buffer to normalize concentration. Next, Pierce^TM^ Anti-HA Magnetic Beads (Thermo Fisher Scientific^TM^) were normalized to concentration and used to IP HA-RING constructs according to manufacturer instructions. Proteins were eluted at 95 °C for 5 min in 1×SDS+2%BME sample buffer (normalized to WCL protein concentration). An empty transfection replicate confirmed that no endogenous BARD1 was co-IPed in absence of HA-RING. There is endogenous expression of BARD1 and UBE2D3 across multiple cell types—human kidney and breast (via Broad Institute Genotype Tissue Expression Portal), and HEK293 (via EMBL-EBI expression atlas).

### 2.4. Immunoblotting and Binding Analysis

WCL (10 μL) and co-IP (25 μL) fractions were run on separate 4–20% Mini-PROTEAN^®^ TGXTM Precast Protein Gels (Bio-Rad^®^; Mississauga, ON, Canada) in 1×SDS running buffer, using a Mini-PROTEAN^®^ Tetra Vertical Electrophoresis Cell (Bio-Rad^®^) powered by a PowerPacTM (Bio-Rad^®^) at 70–100 V/0.3 A, and PageRuler Plus (Thermo Fisher Scientific^TM^) as a reference. Protein transfer to nitrocellulose was performed using a Semi-Dry Transfer Apparatus (Bio-Rad^®^) at 20 V for 42 min in Bjerrum buffer, followed by blocking in PBS with 0.1% Tween^TM^-20 (PBS-T) and 5% skim milk powder for 1 h with constant agitation. Membranes were then cut at the 25 kDa marker to separate BARD1 from UBE2D3 and HA-RING, and incubated overnight at 4 °C with constant agitation in appropriate primary antibodies diluted with PBS-T: BARD1 at 1/1000 for co-IP and 1/4000 for WCL (ab245434; Abcam; Waltham, MA, USA), HA at 1/4500 for co-IP and 1/4000 for WCL (ab9110; Abcam), and UBE2D3 at 1/5000 for co-IP and 1/3000 for WCL (H00007323; Abnova, Thermo Fisher Scientific^TM^). Membranes were then washed with PBS-T for 25 min (changing buffer every 5 min), followed by being incubated for 1 h in the dark with constant agitation and diluted secondary antibodies: goat anti-rabbit conjugated to IRDye 680RD at 1/3000 for co-IP and 1/20,000 for WCL (926-68071, LI-CORbio^TM^; Lincoln, NE, USA) and donkey anti-mouse conjugated to IRDye 800CW at 1/3000 for co-IP and 1/10,000 for WCL (926-32212, LI-CORbio^TM^). Membranes were washed with PBS-T for 25 min (changing buffer every 5 min) before imaging on the 700 nm and 800 nm channels of Odyssey^®^ DLx Infrared Imaging System (LI-CORbio^TM^; Lincoln, NE, USA).

The intensity of relevant bands was quantified on Image Studio (LI-CORbio^TM^). WCL blots confirmed presence of endogenous BARD1 (97 kDa apparent weight [70,71,72,73,74,75,76,77,78]) and UBE2D3 (17 kDa) within HEK293T cells. Co-IPed BARD1 band intensity was normalized to HA-RING (13.8 kDa) detection as a measure of binding capacity, which was expressed relative to WT for standardization across replicates. All changes were converted to base-2 logarithmic ratios (LRs) to improve normality for statistical analyses and visual interpretability. On this logarithmic scale, zero corresponded to no change in binding, and each whole-number fold-change increment above or below zero represented a doubling or halving of binding relative to WT, respectively.

Assay discriminatory performance was assessed by testing whether pathogenic control variants displayed significantly reduced binding compared to benign controls (*p* < 0.05): one-sample one-tailed *t*-tests were conducted for comparisons to WT (μ = 0), and Welch’s two-sample one-tailed *t*-tests were used for all other comparisons. A Benjamini–Hochberg (BH) correction was applied to control the false-discovery rate at α = 0.05 over nine tests. Significant VUS binding losses and gains compared to WT and C24R controls were similarly identified, with a BH-correction used to control for α = 0.05 across the multiple tests within each batch (seven within high-/low- and moderate–high-/moderate–low -scoring batches, and nine within the intermediate-scoring batch). Moreover, observing significantly lower binding compared to WT was interpreted as strong functional evidence for pathogenicity (one-tailed one-sample *t*-test, μ = 0, *p* < 0.05). Conversely, observing significantly higher binding compared to C24R was interpreted as strong functional evidence against pathogenicity (Welch’s two-sample one-tailed *t*-test, *p* < 0.05), provided that variant construct median binding was equal to or greater than that of WT.

### 2.5. Evidence Evaluation

The relationship between in silico and in vitro results was modelled using simple linear regression to assess predictive robustness. Statistical significance of the association was assessed by converting the coefficient of determination (R^2^) to a Pearson correlation coefficient (r), and calculating the two-tailed *p*-value based on the degrees-of-freedom (*df = n_variants_*−2). Correlation was considered significant at *p* < 0.05. The computational algorithm decision threshold was then shifted to the output that best separated variants that gained versus lost BARD1 binding relative to WT empirically, and accuracy on predicting binding loss was assessed.

Similar HA-RING normalization, WT standardization, and statistical comparisons were conducted for co-IPed UBE2D3 across the high- and low-scoring batch, selected as a representative E2 model given its well-characterized broad activity. Linear regression was then used to model the relationship between BARD1 and downstream UBE2D3 binding, with the corresponding R^2^ used to test for correlation significance (*p* < 0.05). Variant scores from the BARD1 binding assay were also compared with HDR activity measurements reported by Starita et al. in 2018, with the corresponding linear regression model R^2^ used to test for correlation significance (*p* < 0.05) [39]. This multiplexed assay was conducted in yeast cells expressing variant BRCA1, and quantified repair to a DSB-damaged green fluorescent protein on a continuous scale (where 0 = no activity, 1 = WT activity, and >1 = activity gain). For variants lacking an individual HDR measurement, the average activity of all other variants at the corresponding residue with available HDR data was substituted.

### 2.6. Variant Classification

Additional variant data was used to inform conclusions on biochemical and structural effects, including the following: existing ClinVar evidence, spatial context from the BRCA1:BARD1 RING heterodimer structure (PDB:1JM7), biochemical insights based on IMGT physicochemical amino acid groupings [79], and predictive annotations from CUPSAT and MutPred2. Potential in vivo mechanisms of pathogenicity not detectable in vitro were also explicitly evaluated: motif analysis predicted effects on NES-mediated localization [80,81], post-translational modification sites were identified on Biomics dbPTM [60], and probability of splicing effect (Δ, reported between 0 and 1) was predicted by Pangolin and SpliceAI. ACMG-AMP recommendations were used to assess the overall evidence for each variant and recommend updated classifications [34].

## 3. Results

### 3.1. Six-Feature Classifier Model Produces Supporting Evidence for 70% of BRCA1 RING VUS

All BRCA1 RING missense variants identified on ClinVar were scored across 52 computational VEPs (Supplementary Table S1), and the average of 21 MolecularFeaST performance metrics was used to rank these features on their ability to discern between pathogenic and benign training variants (Supplementary Table S2; Supplementary Figure S1a). Ensemble features generally performed better than those considering a single aspect of substitution. Specifically, MSA algorithms tended to overpredict pathogenic variants, with models incorporating phylogenetics and deeper sequencing depths offering some improved specificity.

PhyloP 100-vertebrate (20th ranked VEP) represented the inflection point of diminishing performance returns; accordingly, the top 19 features progressed to the next stage of selection (Supplementary Table S2). Ten of these features were removed due to redundancy with others on the list: Mutpred, PROVEAN, VEST4, FATHMM-XF, M-CAP, Eigen, MVP, and DEOGEN were used as features in REVEL and/or MetaRNN training; MutationAssessor was used in Condel training; and BayesDel without allele frequency was redundant to BayesDel with allele frequency.

Training variant data across the nine top-scoring and non-redundant features was input to MATLAB^®^ CLA. All available classifier models (*n =* 32) were built with these features, and average training accuracy was evaluated (Supplementary Table S3). The lowest-ranked feature was then removed, models were rebuilt, and accuracy was reassessed. Models using the top six features (REVEL, Align-GVGD, BayesDel, MetaRNN, Condel, and EVE) achieved the highest average training accuracy (96.5%), informing feature selection for the final BRCA1 RING model.

The majority (18/32) of the models trained on these six features classified both training and test variants with 100% accuracy. Considering the corresponding t-SNE plot showed well-defined separation between pathogenic and benign training variants (Supplementary Figure S1b), the six-feature linear support vector machine (LSVM) was selected. This algorithm offered an efficient (~1100 observations per second) and generalizable model to rank VUSs on a continuous output range based on their distance from a linear decision hyperplane in six-dimensional feature space, enabling detailed prioritization for in vitro analysis. Pathogenic and benign LSVM outputs demonstrated significant separation (*p* < 10^−47^). The six features also appear to discern between two pathogenic variant groups, as 21/24 zinc-coordinating residues and 6/14 non-coordinators clustered together on the t-SNE plot.

To further test the algorithm, a modified LSVM that omitted Condel and EVE (which only enabled predictions for a small subset of proteins) was used to evaluate zinc-finger domain variants across the proteome. The average accuracy on correctly predicting the identity of 721 variants across seven domain types was 85.9%: ZZ (95.8% on 24 variants), PHD (90.7% on 385), C2HC (89.5% on 57), non-BRCA1 RING (84.1% on 151), CXXC (77.8% on 45), TAZ (59.3% on 27), and C2H2 (56.3% on 32) (Supplementary Table S4).

LSVM predictions on VUSs were generally consistent with basic protein and specific BRCA1 RING dynamics (Supplementary Table S5): 12/14 zinc-coordinating residue variants were predicted as the least-tolerable, hydrophilic substitutions at the BARD1 interface and sterically rigid residues that orient zinc-coordinators were predicted-pathogenic, while other residues demonstrated a wider range of tolerance depending on the biochemical conservation of substitution (with phenylalanine and proline generally predicted as the least tolerable substitutions).

**Figure 1:**
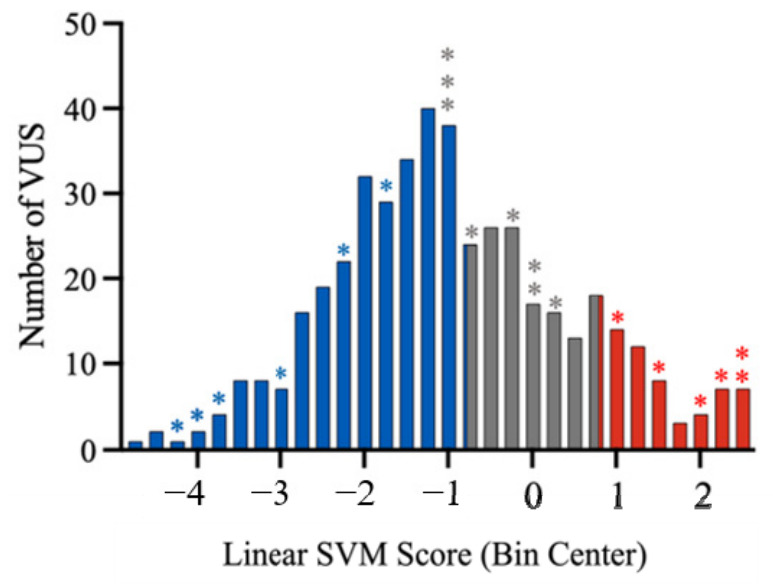
LSVM Scores Predict VUS Pathogenicity. VUS (*n =* 459) LSVM outputs were binned, plotted, and overlaid with red [0.832, 2.466] and blue [−3.799, −0.863] to represent the 95% confidence intervals of known pathogenic and benign outputs, respectively; grey represents the indeterminate range (−0.863, 0.832). Asterisks denote bins from which high/moderately high (red), low/moderately low (blue), and intermediate (grey) scoring variants were selected.

Training and test set LSVM outputs approximated output ranges expected for pathogenic (LSVM > 0.832) and benign (LSVM < −0.863) variants with 95% confidence, with an undefined output range between −0.863 and 0.832 (Supplementary Table S5). The majority (70.2%) of VUSs scored within the 95% confidence interval of pathogenic (58/459) and benign (264/459) variants, providing supporting evidence for and against pathogenicity according to the study-defined criteria, respectively (Figure 1). Within these evidence ranges, predicted-pathogenic VUSs followed a somewhat bimodal score distribution, while benign scores had a wider and right-skewed range.

### 3.2. LSVM 84% Accurate in Predicting BARD1 Binding Loss In Vitro

VUSs were prioritized for in vitro analyses to capture structural diversity, and LSVM outputs across five score categories. The high-scoring group included C27R (79T>C, LSVM = 2.415), H41P (122A>C, 2.410), and C24G (70T>G, 2.285). The moderately high-scoring group included F43C (128T>G, 1.920), L82P (245T>C, 1.430), and L73R (218T>G, 1.027). The intermediate group included I68T (203T>C, 0.298), L86R (257T>G, 0.065), L30S (89T>C, 0.030), P25A (73C>G, −0.263), I15T (44T>C, −0.637), Q54P (161A>C, −0.884), K20Q (58A>C, −0.963), and Q12P (35A>C, −1.005). The moderately low-scoring group included A17D (50C>A, −1.767), N53T (158A>C, −2.330), and D40N (118G>A, −2.876). The low-scoring group included N16S (47A>G, −4.33), C91R (271T>C, −3.96), and E100D (300G>T, −3.65).

**Figure 2:**
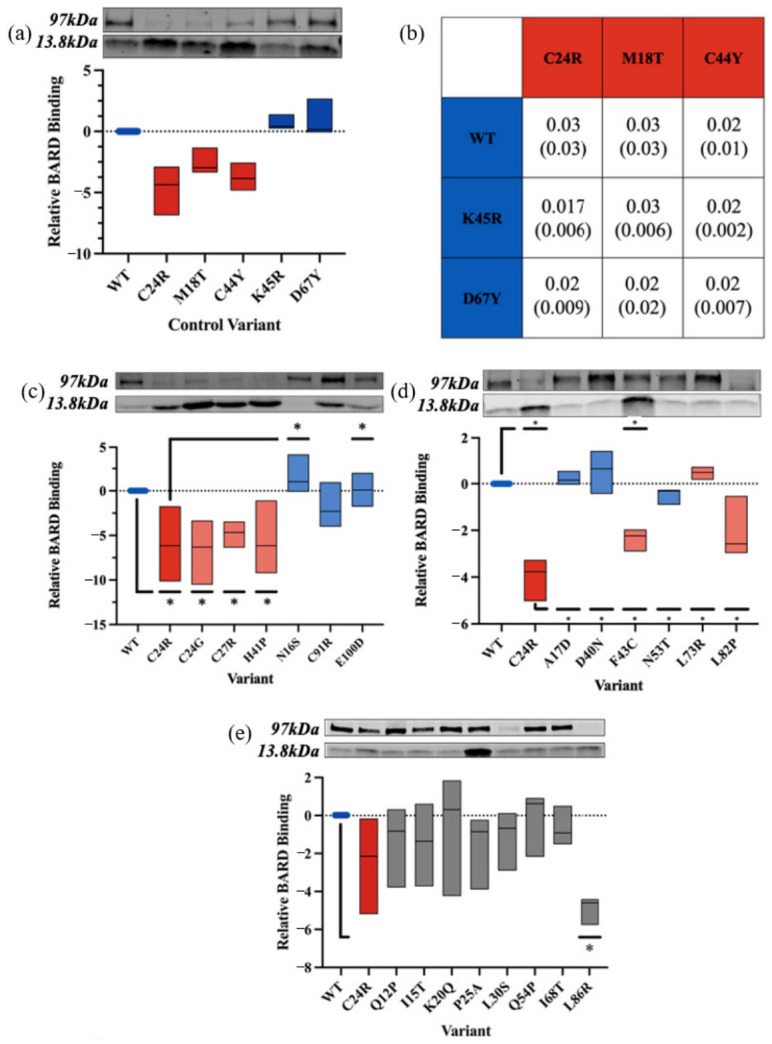
BARD1 Binding Results. Each bar represents the log_2_-transformed BARD1 binding range (measured as ratio of BARD1:HA-RING detection) relative to WT across all replicates with lines denoting the maximum, median, and minimum binding measurements. Colours indicate variant class: dark red and blue represent pathogenic and benign controls, respectively; light red and blue represent VUSs that were high-/moderately high- and low-/moderately low-scoring on LSVM, respectively; and grey represent intermediate-scoring variants. Immunoblotted BARD1 (97 kDa) and HA-RING (13.8 kDa) from one replicate of each batch is shown. (**a**) Control variant BARD1 binding was plotted over three cell-culture replicates, with (**b**) BH-adjusted *p*-values (α = 0.05; unadjusted values in brackets) for pathogenic–benign comparisons summarized. (**c**) High- and low-scoring VUS BARD1 binding plotted over four replicates, (**d**) moderately high- and moderately low-scoring VUS binding plotted across three replicates, and (**e**) intermediate-scoring VUS binding plotted across three replicates using WT and C24R as controls. BH-corrected significance from one-tailed *t*-tests (α = 0.05) is denoted as * *p* < 0.05. Whole blots can be found in Supplementary Figure S2.

Endogenous BARD1 (detected at 97 kDa [70,71,72,73,74,75,76,77,78]) co-IPed from mammalian cell lysate was normalized to the HA-RING construct (13.8 kDa) intensity as a measure of binding capacity, and expressed as a log_2_-ratio (LR) relative to WT for analysis (Figure 2). Significant binding deviations from WT or C24R controls were interpreted as strong functional evidence for and against pathogenicity, respectively (*p* < 0.05). The evidence against pathogenicity was contingent on variant median binding being equal to or greater than that of WT.

Across three biological co-IP replicates, controls defined exclusive ranges of benign (+2.7 to −0.084 LR) and pathogenic (−1.3 to −6.9 LR) BARD1 binding impacts (Figure 2a). All three pathogenic variants demonstrated significantly reduced binding compared to each benign control (*p* < 0.05) after applying BH-correction for nine comparisons (α = 0.05), confirming the functional evidence expectation that variants of opposite classes exhibit significantly different binding (Figure 2b). Notably, zinc-coordinating cysteine substitutions caused a greater median impact than the hydrophobic interface variant M18T. While pairwise control comparisons were significant, the precision was limited between variant replicates and across batches (C24G demonstrated a 1.1- to 1100-fold binding decrease) (Figure 2c–e).

High-/moderately high- and low-/moderately low-scoring VUSs mostly agreed with computational predictions (Figure 2c,d). Of the six high-/moderately high-scoring VUSs, five lost BARD1 binding across all replicates, with four demonstrating significant reductions compared to WT and satisfying study-defined thresholds for strong pathogenic evidence (C24G, C27R, H41P, and F43C). L82P-associated binding losses were variable and did not represent a significant decrease from WT, resulting in indeterminate functional evidence. The moderately high-scoring L73R consistently demonstrated a binding gain that was significantly higher than the C24R activity over three replicates, yielding contradictory strong benign evidence.

Also consistent with strong benign evidence, four low-/moderately low-scoring VUSs (E100D, N16S, A17D, and D40N) exhibited a median increase in binding that was significantly higher than C24R. While three of these variants demonstrated reduced BARD1 binding on at least one replicate, these observations corresponded to the upper-quartile of pathogenic control behaviour (−1.3 to −2.9 LR) and the range of binding that was undefined with tested controls (−0.084 to −1.3 LR). Moderately low-scoring N53T demonstrated significantly greater activity than C24R, but evidence was indeterminate considering all three replicates represented a binding loss within the undefined range. C91R also failed to yield functional evidence since binding was not significantly different compared to either control over four replicates. The activity of this low-scoring variant corresponded to the upper-three quartiles of pathogenic control behaviour (−1.3 to −6.1 LR) on three of the four replicates.

Intermediate-scoring VUSs generally demonstrated mixed binding gains and losses across three biological replicates, with L86R and P25A as notable exceptions that consistently reduced binding (Figure 2e). L86R exhibited a relatively narrow activity range that was significantly lower than WT, consistent with strong pathogenic evidence. Comparatively, the P25A-associated perturbations were minor and variable, and failed to reach significance compared to WT, yielding indeterminate functional evidence. The remaining intermediate-scoring variants demonstrated both binding gains and losses, with a modest median increase (K20Q and Q54P) or decrease (I68T, L30S, I15T, and Q12P) relative to WT that was not significantly different from controls and did not yield classification evidence.

Notably, differences between the HA-RING intensity persisted across replicates and batches, with variant constructs that demonstrated reduced BARD1 binding generally exhibiting a more intense HA signal intensity. WCL analyses demonstrated variations in HA-RING intensity consistent with those observed across co-IPed results.

**Figure 3:**
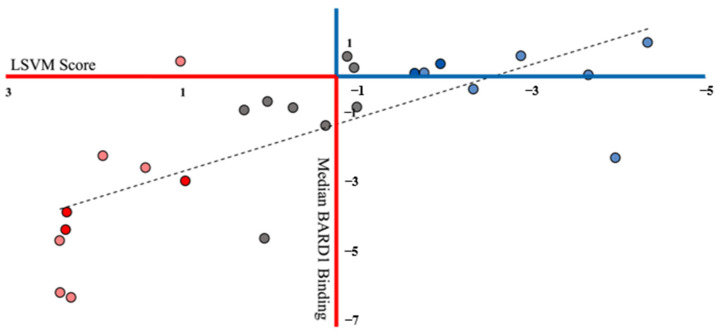
Comparing LSVM Output to BARD1 Binding Results. Median BARD1 binding plotted against LSVM score, with axes coloured to indicate score directionality (red/pathogenic = high LSVM and BARD1 binding loss; blue/benign = low LSVM and BARD1 binding gain). The vertical axis intercept was positioned at x = −0.76 to reflect the empirical decision threshold that maximized accuracy for separating variants that gained versus lost binding activity relative to WT. The black dashed line represented the corresponding linear regression model (y = −0.77x − 1.9, df = 23, R^2^ = 0.53). Datapoint colours indicate variant class: dark red and blue represent pathogenic and benign controls, respectively; light red and blue represent VUSs that were high-/moderately high- and low-/moderately low-scoring on LSVM, respectively; and grey represents intermediate-scoring variants.

Plotting median BARD1 binding against LSVM output visualized the algorithm accuracy on predicting the in vitro impact (Figure 3). Adjusting the decision threshold to −0.76 (midpoint between I15T and Q54P outputs) was the optimal value to discern between variants that empirically lost and gained BARD1 binding relative to WT (84% accuracy). This threshold offered a 93% sensitivity (L73R falsely predicted to lose activity) and 73% specificity (Q12P, N53T, and C91R falsely predicted to gain activity). Further supporting the predictive robustness, a significant correlation was observed between the LVSM score and median BARD1 binding across all variants assayed in vitro (*p* = 0.00001). The relationship was modelled by linear regression: BARD1 binding = −0.77·(LSVM output) − 1.9.

### 3.3. BARD1 Binding Correlates with Downstream BRCA1 RING Functions

**Figure 4:**
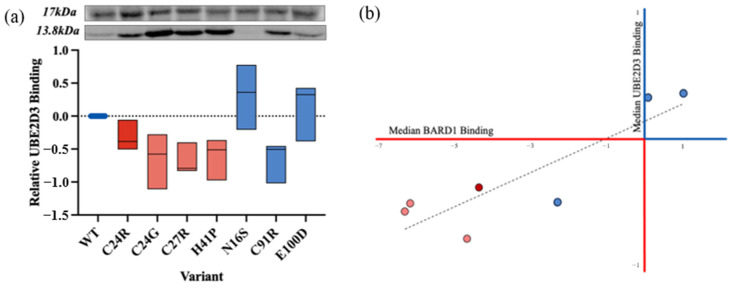
Relationship between BARD1 and UBE2D3 Binding. (**a**) High- and low-scoring VUS UBE2D3 binding was plotted over three replicates, measured as the ratio of 17 kDa UBE2D3 to 13.8 kDa HA-RING detection on immunoblot, with an example shown. Each bar represents UBE2D3 binding range expressed as an LR relative to WT, with lines denoting maximum, median, and minimum binding measurements. Colours correspond to variant class: dark red and blue represent pathogenic (C24R) and benign (WT) controls, respectively; and light red and blue represent high- and low-scoring VUSs, respectively. No variants demonstrated significantly different binding compared to controls on one-tailed *t*-testing after BH-correction (α = 0.05; *p* > 0.05). Whole blots can be found in Supplementary Figure S2. (**b**) Median UBE2D3 binding plotted against median BARD1 binding, with axes coloured to indicate score directionality (red/pathogenic = UBE2D3 and BARD1 binding loss; and blue/benign = UBE2D3 and BARD1 binding gain). The black dashed line represents the linear regression model (y = 0.14x − 0.14, df = 5, R^2^ = 0.8).

UBE2D3 (detected at 17 kDa) binding was quantified as an LR relative to WT on three replicates of the high- and low-scoring VUS batch (Figure 4a). VUS UBE2D3 binding generally mirrored the trends observed with BARD1 binding, but with smaller magnitudes of change that were not significantly different from controls (*p* > 0.05) after BH-correction for seven one-tailed *t*-test comparisons (α = 0.05). The pathogenic control also failed to demonstrate significantly lower binding than WT constructs. However, there was 100% concordance between the direction of results: C24R, C24G, C27R, H41P, and C91R demonstrated median BARD1 and UBE2D3 binding losses; and N16S and E100D constructs were associated with an increase in both BARD1 and UBE2D3 binding.

**Figure 5:**
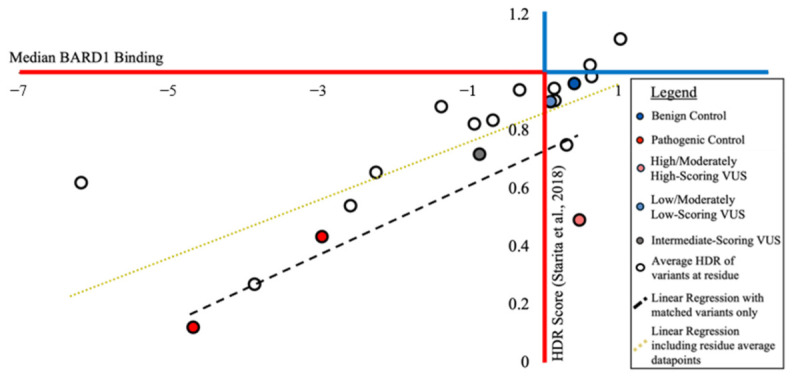
Relationship Between BARD1 Binding and HDR Function. HDR activity [39] plotted against median BARD1 binding, with axes coloured to indicate score directionality (red/pathogenic = HDR and BARD1 binding loss; blue/benign = HDR and BARD1 binding gain). Filled dots represent variants with a specific HDR score (dark red and blue represent pathogenic and benign controls, respectively; light red and blue represent VUS that were high-/moderately high- and low-/moderately low-scoring on LSVM, respectively; grey represents intermediate-scoring variants), while empty dots represented the average HDR score of all variants at residues where data for the specific variant of interest was unavailable. The black dashed line represented a linear regression model with only the datapoints for specific variants that had results on both assays (y = 0.12x + 0.76, df = 4, R^2^ = 0.69), and the yellow dashed line represented the linear regression model that included all datapoints (y = 0.10x + 0.86, df = 18, R^2^ = 0.57).

*HDR Function*—BARD1 co-IP results were compared to HDR activity data reported by Starita et al. (Figure 5) [39]. A significant correlation between measurements was observed across six variants with both datapoints available (*p* = 0.04), and when including averaged HDR activities at 14 additional residues (*p* = 0.0001).

### 3.4. BARD1 Binding Results Are Rationalized by BRCA1 RING Structure and Biochemistry

**Figure 6:**
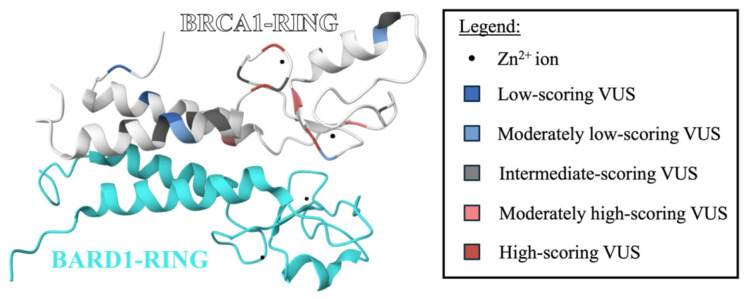
Structural Context of VUSs. VUSs assayed in vitro mapped to PDB:1JM7 model of the zinc-coordinated BRCA1 RING and BARD1 RING heterodimer.

The structural and biochemical context of VUSs within the BRCA1:BARD1 heterodimer was consistent with the effects on binding observed in vitro (Figure 6; Supplementary Table S6). Substitutions at zinc-coordinating residues (C27R, H41P, and C24G) resulted in a loss of coordination, dependent BARD1-binding, and the transient enhancement in UBE2D3 binding. F43C also impedes zinc-coordination, possibly through the loss of bulky steric effects that orient H41 and C44, or the introduction of a new coordinating cysteine. Distinctly, the L86R and L82P substitutions in helix #3 directly disrupt BARD1 binding by reducing the interface hydrophobicity and introducing helix-disrupting conformational rigidity, respectively.

The structural and biochemical context of E100D (hydrophilicity maintained, adjacent to helix #3) and N16S (polarity maintained, solvent-exposed, and oriented away from BARD1 interface in helix #1) supports their benign impact on the BARD1 interface and downstream UBE2D3 binding. Similarly, the structurally conservative D40N binding loop alteration, and hydrophilic A17D substitution adjacent to the hydrophobic interface do not meaningfully impact coordination or heterodimerization. The observed L73R binding retention is consistent with its position in an unstructured region between the binding loop and helix #3, but MutPred2 computed a 6% probability that this substitution introduces a distinct mechanism of pathogenicity by promoting Q74 cyclization, resulting in the decreased degradation and increased aggregation of BRCA1.

Intermediate BARD1 binding effects observed with the remaining variants were likely explained by the minor deoptimization of the hydrophobic interface via direct changes (C91R, Q12P, K20Q, and I15T) or zinc-positioning impacts (P25A effects C24, L30S effects C27, I68T effects C64, and Q54P and N53T effect helix #2 between binding loops). Notably, MutPred2 computed a 27% probability that the loss of a rigid backbone residue in P25A induces a helical gain within the first zinc-binding loop, potentially causing a more severe effect on zinc-mispositioning than other non-coordinating binding loop variants. This exceptional effect of P25A on zinc-positioning would corroborate the consistent loss of BARD1 binding observed across all replicates.

Alternate mechanisms of variant pathogenicity undetectable by in vitro cDNA co-IP assays were assessed (Supplementary Table S6). The effects on post-translational processing were considered unlikely as no VUS resided within well-characterized enzyme motifs or at modified residues identified on dbPTM [60]. Since L86R and L30S result in the loss of a hydrophobic residue within the NES exportin-recognition motif, these variants may cause aberrant nuclear accumulation during the early *S* phase in vivo [80,81]. Comparatively, C24G, P25A, L82P, and C91R fall at noncritical residues within NES and likely do not perturb localization. Pangolin and SpliceAI predict that only four VUSs have a possible (0.2 < ∆ < 0.5) impact on splicing at distal sites: L73R (splice loss at F79), I68T (loss at Q81, gain at S59), L86R (gain at K110 and I42), and C91R (gain at E149 and K70).

## 4. Discussion

Combining the computational (LSVM) and functional (BARD1 co-IP) evidence described in this study warrants the reclassification of four VUSs to clinically actionable categories and under current ACMG-AMP guidelines (Table 1) [34]. H41P now meets the criteria for the likely pathogenic classification with corroborating strong functional and supporting computational evidence, combined with moderate evidence for being located at the same-residue as known pathogenic mutations (H41D/Y/N). While C27R and C24G also meet this criterion, these variants were classified as likely pathogenic by another group during the course of this study—with C24G satisfying higher standards for pathogenic classification considering its absence from gnomAD (moderate pathogenic evidence) and presence in one case of sporadic breast cancer (supporting pathogenic evidence from phenotypic connection) [82]. Conversely, N16S, A17D, and E100D met the standards for the likely benign classification with corroborating computational and functional evidence.

Nine variants received meaningful evidence in this study, despite not reaching ACMG-AMP standards for reclassification. While F43C obtained consistent computational and functional evidence, the variant does not meet the likely pathogenic classification standards without additional moderate evidence. D40N also received corroborating evidence in this study, but contradictory moderate (gnomAD absence) and supporting (variant observed in one patient with HBOC phenotype) pathogenic evidence prohibits a likely benign designation. Seven VUS (Q12P, L86R, L82P, N53T, Q54P, K20Q, and C91R) received either computational or functional evidence, while the other form of evidence was indeterminate. Although these variants currently remain of uncertain significance, forthcoming ACMGv4.0 guidelines will enable the VUS-high (F43C, L86R, and L82P) and VUS-low (D40N, Q54P, C91R, N53T, K20Q, and Q12P) classifications to denote partial evidence for and against pathogenicity, respectively, which may prove useful for providing some clarity to VUS screening results, and guide decisions regarding surveillance [83].

Computational and functional approaches yielded no evidence for four variants, which will remain as VUSs with no clinically meaningful insight (L30S and I15T), or with moderate same-residue evidence in favour of pathogenicity (P25A and I68T). Finally, L73R yielded conflicting strong benign and supporting pathogenic evidence and remains classified as a VUS; however, the overall weighted evidence leans against pathogenicity.

The LSVM algorithm achieved a perfect and significant (*p* < 10^−47^) separation between all known BRCA1 RING pathogenic and benign variants. Accordingly, thresholds defined on the 95% confidence intervals of training and test variants provided robust supporting evidence for the majority (322/459) of ClinVar-documented VUSs within BRCA1 RING (Figure 1; Supplementary Table S5). While the limited number of classified BRCA1 RING variants constrained rigorous algorithm testing, a modified LSVM did achieve 85.9% accuracy across 721 variants in other zinc-finger domains (Supplementary Table S4). However, because two features were removed to enable genome-wide predictions, these variants could not be interpreted as a true test set. Despite their biochemical and structural similarity to the BRCA1 RING domain, predictive accuracy was reduced in the other proteins bearing zinc-finger domains. This suggests that the model’s strong performance on BRCA1 RING is not attributable solely to structural and biochemical similarities common to zinc-finger domains but also reflects the integration of RING domain-specific properties captured by the selected features. Nevertheless, the relatively high accuracy on diverse zinc-finger domains provides some support for the algorithm generalizability to unseen variants. Moreover, the linear decision boundary offers inherent advantages of generalizability, efficiency, and interpretability [84]. Future work is required to elucidate the biological and mechanistic basis of these observed differences across zinc-finger domains.

The LSVM also satisfies specific ACMG-AMP guidelines for computational evidence. Foremost, domain-specific training aligned with the requirement to compile multiple relevant VEPs for a single line of evidence [34]. The selected features performed accurately on BRCA1 RING and had biological relevance to domain-specific dynamics, further supporting the generalizability to VUS predictions (Supplementary Figure S1, Supplementary Table S2). The low specificity of MSA-based VEPs likely reflects pathogenicity thresholds calibrated to proteome-wide conservation patterns, which are incompatible with the high baseline conservation of BRCA1 RING across distantly related orthologs. Multiple benign *BRCA1* variants exist at residues that are perfectly conserved across evolution—these minor disruptions experience strong negative selection over macroevolutionary time, but the impact on individual cancer risk is often negligible [85,86]. Accordingly, ensemble features that incorporated additional structural, biochemical, and allele frequency context generally performed better on BRCA1 RING variants and constituted most of the final algorithm. MSA-based PROVEAN was also included in the LSVM algorithm as it utilizes a deep ~300-sequence alignment and considers conservation in a local window, which seemingly provides meaningful information to distinguish benign variation at otherwise perfectly conserved residues [87]. This task remains challenging for leading deep-learning MSA-based VEPs trained across the proteome, such as Google DeepMind’s AlphaMissense [88]. AlphaMissense achieved 100% sensitivity and 72% specificity on BRCA1 RING. Although AlphaMissense was not yet available during the development of our algorithm, we subsequently utilized it as a gold standard comparator to our model. The superior predictive performance achieved by the LSVM on BRCA1 RING variants suggests that the incorporation of structural, biochemical, and allele frequency context may offer advantages over proteome-wide protein language models when evaluating variants within specialized functional domains.

LSVM VUS output distribution reflected the expected variant trends (Figure 1). The predominance of predicted-benign variants is consistent with the enrichment in neutral variation expected in the human genome [89,90]. The bimodal predicted-pathogenic score distribution, together with the visual separation of two pathogenic training variant groups in t-SNE space (Supplementary Figure S1b), suggests that the LSVM may also reasonably distinguish between pathogenic effects arising from the loss of zinc-coordination versus a direct impact to the BARD1 binding interface. These distinct effects were generally observed in vitro (Figure 2), and LSVM outputs were significantly correlated to BARD1 binding activity overall (*p* = 0.00001; Figure 3). This provided further validation that that the algorithm was generalizable to the full range of BRCA1 RING variant impacts—the highest and lowest outputs corresponding to the greatest losses and gains, respectively, with intermediate scores reflecting more subtle binding reductions (Figure 3). Accordingly, the model was effective for prioritizing high- and low-scoring VUSs likely to yield consistent and well-defined BARD1 binding results, thereby maximizing the low-throughput assay value, and supporting the clinical use of robust computational models to flag de novo sequenced variants for immediate in vitro analysis. Additionally, as variant classification trends towards personalized risk-assessments, a reliable non-binary evidence system may enable finer discrimination between the severity of pathogenic mutations, and benign variants with clinically meaningful hyperactivity [91].

Significant differences were observed between each pathogenic and benign control pair, validating that biochemically and structurally diverse pathogenic and benign BRCA1 RING variants converge on the loss and retention of BARD1 binding, respectively (Figure 2a,b). Activity ranges were well-separated, with future work required to confirm whether binding losses between −0.084 and −1.3 LR sufficiently abrogate BRCA1:BARD1 activities to meaningfully increase the cancer risk or represent a tolerable benign impact that preserves sufficient downstream tumour suppressive activity. This would specifically help clarify the results of N53T, C91R, and P25A. Likewise, the biological significance of VUSs exhibiting higher median binding than benign controls (N16S, D40N, Q54P, and L73R) remains unresolved, as they may reduce the cancer risk, contribute to other pathologies (e.g., Alzheimer’s Disease), or be buffered by BRCA1 homeostatic regulation and result in no change to cancer predisposition [92,93].

This low-throughput BARD1 co-immunoprecipitation assay offers potential advantages over commonly used reference assays. Physicians report relatively lower confidence in interpreting results from multiplexed approaches [35,94,95]. Findlay et al.’s HAP1 viability assay measured a less-direct proxy to the tumour-suppressive function of BRCA1 compared to a BARD1 co-IP [35]. Offering a more direct comparison, Clark et al.’s co-IP and mammal approaches quantified the binding between exogenous BARD1 and BRCA1 RING constructs, which may be less representative of the native interaction compared to the endogenous-BARD1 model described here [49]. Nevertheless, the perfect separation of all control variants tested in both the exogenous- and endogenous-BARD1 co-IP models work together to increase our confidence that BARD1 binding is a robust and established predictor of pathogenicity, as per ACMG-AMP guidelines [34]. Moreover, nine of the ten of the variants that yielded functional evidence from this study showed concordant results in both reference assays. L73R was the exception, supporting that this substitution introduces a unique pathogenicity mechanism not readily detected by endogenous-BARD1 co-IP. While this contradiction was flagged by MutPred2 in the assay workflow, it highlights the value of consulting multiple complementary in vitro reference models that compensate for specific limitations in recapitulating in vivo tumour suppression. Highlighting another potential discrepancy between in vitro conditions and the true physiology, the detection of pathogenic-behaving HA-RING constructs was generally higher on the immunoblot. Importantly, the WCL analyses showed HA-RING protein expression variations consistent with co-IP results. This concordance indicates that these variations are not artifacts specific to the co-IP but may be reflective of underlying expression differences across constructs. Therefore, these observations may reflect biologically meaningful pathogenic construct behaviour that could be exploited for a simplified future assay. Possible mechanisms include decreased degradation, increased synthesis, or the loss of the complex formation increasing accessibility to the HA-tag (facilitating IP) and NES (resulting in nuclear exclusion and the improved recovery of protein during lysis). Poor reproducibility was also noted as a potential assay limitation, but was not entirely unexpected considering previous work has shown that some BRCA1 variant effects on folding and activity are sensitive to environmental conditions such as temperature [96,97], and chaperone abundance [98]. Accordingly, differences between the cellular (e.g., passage number), experimental (e.g., reagent age), and ambient (e.g., temperature) conditions likely contributed to the observed variability. Interestingly, tuning conditions to recapitulate distinct cellular environments may enable the tissue-specific interpretation of variant pathogenicity. As with all BRCA1 assays, the ACMG-AMP criteria for directly measuring the tumour-suppressive function were not satisfied. However, the literature evidence and significant correlations between BARD1 binding and downstream activities support the interaction as an obligate upstream event and a reliable proxy for the overall domain function, capturing both the proximal (UBE2D3 binding, *p* = 0.01; Figure 4) and distal (HDR activity, *p* = 0.04; Figure 5) effects. Overall, the association between BARD1 binding and pathogenicity appears to reflect a mechanistic link to tumour suppression rather than a spurious correlation. Furthermore, the high level of concordance between the functional results from our BARD1 assay and those reported in the reference assays from Starita et al., Findlay et al., and Clark et al. further validates its biological relevance (Supplementary Table S7).

As BRCA1 functions as a multi-domain scaffold protein with evidence of coordinated domain contributions and architectures, variants external to the RING domain may indirectly influence RING stability or activity through effects on protein folding, and the conformational organization and assembly of BRCA1 complexes. Such effects may not be fully captured through domain-centric assays, highlighting the value of full-length BRCA1 functional systems for a more comprehensive assessment of variant impacts on RING structure and activity.

Compared to global upstream BARD1 binding, assaying a downstream phenotypic state of *BRCA1*-deficiency may also circumvent the need for directly measuring tumour-suppressive function. As synthetic lethal therapies exploit PARP dependence, inhibitor assays may provide evidence for both variant classification and drug sensitivity [18,67]. The ability to identify both risk and predictive biomarkers would enhance the value of screening programs and may increase the number of women eligible over time. Considering that few patients follow up on predictive test referrals received during diagnostic testing, the combined delivery is especially relevant [99,100]. Similarly, identifying variants that produce long-molecule genomic scars associated with compensatory break-induced replication repair upregulation may serve as evidence for pathogenicity and therapy responsiveness [101]. Expanded-coverage next-generation screens may detect these scars in vivo, which would be of particular value in stage II–III BC by enabling immediate targeted therapy initiation. Given the strict requirement for cells to activate compensatory pathways to survive the BRCA1 loss of heterozygosity, many other leverageable synthetic lethal relationships likely exist. Most of the BRCA1 synthetic lethality research has been conducted on the BRCT domains, with future work required to confirm that C’-terminal synthetic lethal relationships hold on the RING domain (e.g., CHK1, WEE1, ATR, Pol-θ, and histone deacetylase) [102,103,104].

## 5. Conclusions

The domain-specific LSVM algorithm and BARD1 binding assays aligned with ACMG-AMP guidelines and yielded supporting evidence for 322 VUSs and strong corroborating evidence for 9 VUSs, which warranted the reclassification of 4 variants. Once updated in ClinVar, this will immediately enhance the clinical decision-making for carriers of these variants. This study also highlights the promise of domain-specific workflows for generating robust evidence for missense VUSs.

## Figures and Tables

**Table 1:** Summary of VUS evidence and updated classifications.

VUS	Supporting Computational	Strong Functional	Moderate Same- Residue	Moderate GnomAD	Supporting Phenotype	Updated Classification
C24G	P	P	P	P	P	Pathogenic **
C27R	P	P	P			Likely Pathogenic **
H41P	P	P	P			Likely Pathogenic
F43C	P	P				VUS-High ***
L86R	?	P *				VUS-High ***
I68T	?	? *	P			VUS-High ***
P25A	?	? *	P			VUS-High ***
L82P	P	?				VUS-High ***
L30S	?	? *				VUS
I15T	?	?				VUS
L73R	P	B *				VUS-Low ***
Q12P	B	?				VUS-Low ***
K20Q	B	?				VUS-Low ***
N53T	B	?				VUS-Low ***
C91R	B	? *				VUS-Low ***
Q54P	B	?				VUS-Low ***
D40N	B	B		P	P	VUS-Low ***
A17D	B	B				Likely Benign
E100D	B	B				Likely Benign
N16S	B	B				Likely Benign

P = pathogenic evidence; B = benign evidence; ? = Indeterminate results. * = alternate mechanism of pathogenicity potentially missed in cDNA co-IP assay. ** = updated by another group during course of this study. *** = classification under ACMGv4.0 guidelines, VUSs under current system.

## Data Availability

BRCA1 (and other zinc-finger domain) variant statistics and data analysed in this study were obtained from ClinVar (https://www.ncbi.nlm.nih.gov/clinvar/, accessed on 1 April 2024 for collecting variants, accessed on 1 April 2026 for statistics). In silico scores were obtained either through the dbNSFP database on the Ensembl Variant Effect Predictor (VEP) web interface (https://www.ensembl.org/info/docs/tools/vep/index.html, accessed on 1 April 2024) or directly from individual in silico tool webpages: BRASS (www.biotoclin.org/predictor/BRCA1/help/, accessed on 1 September 2022), Align-GVGD (agvgd.hci.utah.edu/agvgd_input.php, accessed on 1 September 2022), Shannon Entropy (http://imed.med.ucm.es/PVS/, accessed on 1 September 2022), PON-P2 (https://structure.bmc.lu.se/PON-P2/, accessed on 1 September 2022), SpliceAI and Pangolin (https://spliceailookup.broadinstitute.org/, accessed on 1 September 2022), MutPred2 (https://mutpred.mutdb.org/, accessed on 1 September 2022), and CUPSAT (https://cupsat.brenda-enzymes.org/, accessed on 1 September 2022). The human BRCA1 RING domain sequence used for variant construct generation, along with orthologous sequences for MSAs, were obtained from the NIH (https://www.ncbi.nlm.nih.gov/datasets/gene/672/#orthologs, accessed on 1 September 2022); COBALT was used to construct alignments (https://www.ncbi.nlm.nih.gov/tools/cobalt/cobalt.cgi?CMD=Web, accessed on 1 September 2022). Structural models of BRCA1 RING bound to BARD1 were obtained through Protein Data Bank (PDB) (https://www.rcsb.org/structure/1JM7, accessed on 1 January 2026). Zinc-finger domains outside of BRCA1 were identified using the Hugo Gene Nomenclature Committee index (https://www.genenames.org/data/genegroup/#!/group/26, accessed on 1 January 2026) and UniProt (https://www.uniprot.org/, accessed on 1 January 2026). The endogenous expression of BARD1 and UBE2D3 across multiple cell types was confirmed using The Broad Institute Genotype Tissue Expression Portal (https://gtexportal.org/home/, accessed on 1 September 2025) and EMBL-EBI (https://www.ebi.ac.uk/, accessed on 1 September 2025). Post Translational Modification sites were identified using dbPTM (https://biomics.lab.nycu.edu.tw/dbPTM/info.php?id=BRCA1_HUMAN, accessed on 1 September 2025). The data generated in this study are available within the article and its Supplementary Data Files.

## Supplementary Materials

The following supporting information can be downloaded at: https://www.mdpi.com/article/10.3390/curroncol33070399/s1, Table S1: Training and Test Score Reference; Figure S1: Feature Selection and Model Building Results; Table S2: Feature Selection Reference; Table S3: Model Building Reference; Table S4: Modified LSVM outputs for zinc-finger variants across the proteome; Table S5: LSVM Scores for All ClinVar-Documented BRCA1 RING Variants; Figure S2: Whole Representative Immunoblots from Figure 2 and Figure 4; Table S6: Summary of VUS Properties; Table S7: BRCA1 Functional Assay Evidence from Martin et al., Starita et al., Findlay et al., and Clark et al.

## Abbreviations

The following abbreviations are used in this manuscript:

ACMG	American College of Medical Genetics and Genomics
AMP	Association for Molecular Pathology
BARD1	BRCA1-Associated RING-Domain protein
BC	breast cancer
BME	β- mercaptoethanol
BRCA1	BReast CAncer Type 1 Susceptibility Protein
*BRCA1*	BReast CAncer Gene 1
BRCT	BRCA1 C-Terminal repeats
BH	Benjamini–Hochberg
CLA	classification learner app
Co-IP	co-immunoprecipitation
DSB	double-strand break
E2	ubiquitin-conjugating enzyme
fitCons	fitness consequence
gnomAD	Genome Aggregation Database
HBOC	Hereditary Breast and Ovarian Cancer Syndrome
HDR	homology-directed repair
HEK293T	Human Embryonic Kidney 293 cells expressing SV40 large T-antigen
LR	log_2_ ratio
LSVM	linear support vector machine
MFeaST	Molecular Feature Selection Tool
MSA	Multiple Sequence Alignment
NEB^®^	New England Biolabs^®^
NES	nuclear export sequence
OC	ovarian cancer
PARP	Poly (ADP-ribose) polymerase
PBS-T	phosphate-buffered saline + 0.1% Tween^TM^-20
RING	Really Interesting New Gene domain
SVM	support vector machine
t-SNE	t-distributed stochastic neighbour embedding plot
UBE2D3	Ubiquitin-conjugating enzyme E2 D3
VUS	variant of uncertain significance
WCL	whole-cell lysate
WT	wild-type
